# Two new Oriental species of *Eumorphus* Weber (Coleoptera, Endomychidae)

**DOI:** 10.3897/zookeys.677.10399

**Published:** 2017-05-26

**Authors:** Ling-Xiao Chang, Guo-Dong Ren

**Affiliations:** 1 College of Life Sciences, Hebei University, Baoding 071002, China

**Keywords:** Coccinelloidea, Coleoptera, Lycoperdininae, new species, Oriental Region, taxonomy

## Abstract

Two new species of *Eumorphus* from Asia, *E. falcifasciatus* **sp. n.** and *E. qiujianyuei* **sp. n.** are described and illustrated.

## Introduction

The genus *Eumorphus* was established by Weber (1801) with *Eumorphus sumatrae* Weber, 1801 (= *Erotylus quadriguttatus* Illiger, 1800) as the type species. This genus is classified in the largest subfamily of Endomychidae, Lycoperdininae, the monophyly of which was tested and confirmed by the phylogenetic studies of Tomaszewska (2000, 2005). Robertson et al. (2015) presented a large-scale phylogenetic study for the Cucujoidea, using molecular evidence to rebuild the relationship tree of this superfamily and established one new superfamily, Coccinelloidea, with Endomychidae placed within it. This study further confirmed the monophyly of the subfamily Lycoperdininae and established its sister relationship with the subfamily Epipocinae (Robertson et al. 2015).

Tomaszewska (2005) recognized five generic groups among 38 genera of Lycoperdininae known at that time. The 23 genera known then from the Oriental Region have been classified in four of five generic groups (*Lycoperdina*-, *Amphix*-, *Amphisternus*- and *Eumorphus*-groups). Since then two new genera of Lycoperdininae have been described from the Oriental Region: *Stroheckeria* Tomaszewska, 2006 and *Humerus* Chang & Ren, 2013. Both, indicated by the authors as belonging to *Amphisternus*-group (Tomaszewska 2006, Chang and Ren 2013), sister group of *Eumorphus*-group which includs the genus *Eumorphus* Tomaszewska (2005).

The *Eumorphus*-group includes 14 genera, five of which are distributed in the Oriental Region: *Avencymon* Strohecker, 1971, *Encymon* Gerstaecker, 1857, *Eumorphus* Weber, 1801, *Platindalmus* Strohecker, 1979 and Parindalmus Achard, 1922.

Strohecker (1968) listed 73 species (including subspecies) in his synopsis of the genus *Eumorphus*, of which *E. convexus*, *E. cryptus*, *E. elegans*, *E. eurynotus*, *E. leptocerus*, *E. micans*, and *E. parvus* were described as new species, and *E. austerus indianus*, *E. bipunctatus crucifer*, *E. bipunctatus mirus*, and *E. murrayi carinensis* were introduced as new subspecies. In addition, eleven nominal species were reduced to subspecies: *E. assamensis subsinuatus*, *E. bulbosus arrowi*, *E. coloratus vitalisi*, *E. cyanescens thomsoni*, *E. dilatatus turritus*, *E. eburatus guerini*, *E. fryanus festivus*, *E. fryanus quadripustulatus*, *E. quadriguttatus andamanensis*, *E. quadriguttatus convexicollis*, and *E. sybarita consobrinus*. Subsequently two species were removed from *Eumorphus* and transferred to other genera, *E. calcaratus* Arrow, 1920 to *Platindalmus* (Strohecker 1979) and *E. nanus* Arrow, 1920 to *Indalmus* (Strohecker 1971); and one species was been transferred into *Eumorphus*: *Engonius bicoloripedoides* (Mader, 1955) by Strohecker (1968).

In 2007, Ren and Wang described two new species of *Eumorphus*, *E. dentatus* and *E. letilimarginatus* from China. *Eumorphus* is the largest genus of the subfamily Lycoperdininae and prior to the present study, this genus included 76 species (including subspecies) (Shockley et al. 2009).

During the examination of Endomychidae collected in China and Borneo, two new species were recognized and are described here.

## Materiasl and methods

Type specimens of the new species described here are deposited in the following institutions or private collections:

**MHBU** Museum of Heibei University, Baoding, China

**CCLX** Collection of Lingxiao Chang, Beijing, China

The specimens were examined and described using a Nikon^®^ SMZ800 dissecting microscope. The following measurements were made using a Leica^®^ M205 A dissecting microscope: body length from apical margin of clypeus to apex of elytra; width across both elytra (at widest part); elytral length along suture, including scutellum. The abdomen was boiled in 10% NaOH solution, cleaned, and finally aedeagus was dissected in distilled water. Habitus photos were taken using a Canon^®^ Eos 5D III SLR camera and Canon^®^ MP-E 65mm macro lens. All photographs were modified in Adobe Photoshop^®^ CC 2015.

## Taxonomy

### Eumorphus

Taxon classificationAnimaliaColeopteraEndomychidae

Weber, 1801

Eumorphus Weber, 1801: 31. Type species. Erotylus quadriguttatus Illiger, 1800.
Eumorphoides Guérin–Méneville, 1858: 12. Type species.Eumorphus tetraspilotus Hope, 1832.
Enaisimus Guérin–Méneville, 1858: 16. Type species. Eumorphus quadrinotatus Gerstaecker, 1857.
Haplomorphus Guérin–Méneville, 1858: 18. Type species. Eumorphus bipunctatus Perty, 1831.
Heterandrus Guérin–Méneville, 1858: 26. Type species. Eumorphus confusus Guérin–Méneville, 1857.

#### Diagnosis.

The species of *Eumorphus* are most similar to those of *Platindalmus* and *Gerstaeckerus*. However, *Eumorphus* can be distinguished from these other genera by the following combination of characters: 1) lateral margin of pronotum with a tendency to form irregularly broken lines, inconstant and often asymmetrical; 2) mandible is narrowly chisel-shaped at its apex; 3) elytra with basal margin simple; 4) intercoxal process of mesoventrite with lateral margins subparallel; 5) male femora lacking fringes of long hairs on inner edges (after Tomaszewska 2005).

### Eumorphus falcifasciatus sp. n.

Taxon classificationAnimaliaColeopteraEndomychidae

http://zoobank.org/1F8715EC-E053-4819-829C-54B9A59E769C

Figs 1 , 3

#### Type material.

Holotype, male, Borneo, Sabah, Keningau district, Jungle Girl Camp, 1215 m, 2016-IV-26, Chang L.X. leg (CCLX).

**Figure 1.**
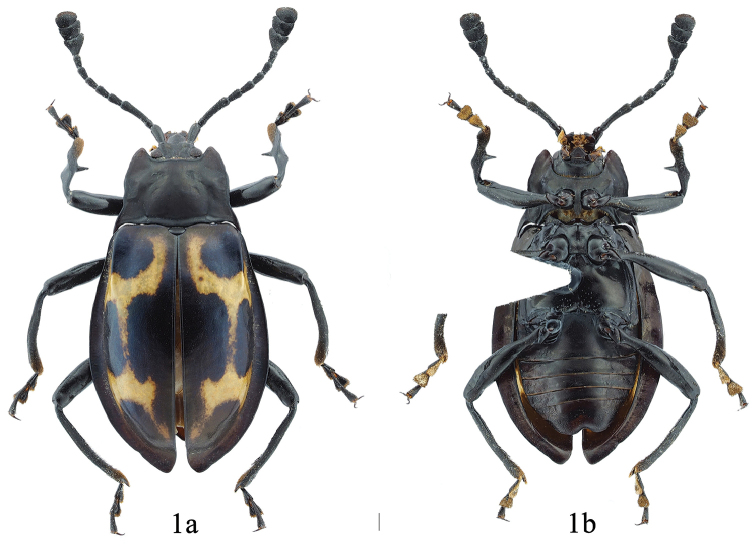
Dorsal and ventral habitus of *E. falcifasciatus* sp. n. male. **a** dorsal view **b** ventral view. Scale bar 1 mm.

#### Diagnosis.

*Eumorphus falcifasciatus* is a very unique species by its colouration, differing from all others in having the anterior elytral maculae falciform and posterior maculae dentate.

#### Description.

Length 16.1 mm. Body pyriform, approximately 1.9 times as long as wide; moderately convex; subopaque. Colour black brown with two yellow maculae on elytra.

*Head*. Antenna composed of 11 antennomeres, long, rather stout, nearly 1/2 body length, with antennomeres 1 and 3–8 distinctly longer than wide; scape approximately 5.5 times as long as pedicel; pedicel very short, nearly as long as wide; antennomere 3 longer than 4–5 combined; antennomere 4 as long as 5; antennomeres 5 slightly longer than 6; antennomeres 6–8 subequal in length; club composed of three antennomeres, very broad, approximately 4.0 times as wide as antennomere 8, moderately flat and compact.

*Thorax*. Pronotum 3.5 mm long, 5.7 mm wide; widest at base; finely, rather densely punctate; lateral and anterior margins narrowly bordered; anterior edge with small stridulatory membrane; sides undulate, abruptly widened basally from 1/4 length; anterior angles produced, rather acute; posterior angles strongly, acutely produced backwards, distinctly curved basally and overlapping most of humeri; disc weakly convex, surface uneven with one large transverse oval raised area posteromedially and two small round raised areas anterolaterally; median furrow absent; lateral sulci shallow, linear, extending to 1/2 pronotal length; basal sulcus weakly sinuate, moderately deep. Prosternal process moderately widely separating the procoxae; gradually widening to apical 1/4, thence abruptly converging towards apex. Mesoventral process nearly quadrate, disc weakly convex, sides subparallel. Elytra 11.8 mm long, 8.3 mm wide; 1.4 times as long as pronotum; 1.5 times as wide as pronotum, sides curved, widest near behind 1/2 length of elytron; lateral flattened margins abruptly widening from basal 1/6 to apex, nearly 1/5 of elytral width; sides distinctly converging from apical 1/3 towards apex; finely, densely punctate; humeri weakly prominent. Each elytron with two large irregular maculae. Anterior elytral macula falciform, occupies about 4/5 of elytral width and 2/3 of elytral length, outer sides touching elytral lateral margin, inner margin of macula placed closely to elytral suture. Posterior macula crown-shaped, located at apical 1/3, its anterior margin tridentate, posterior margin widely emarginate medially. Protibiae slender basally, abruptly widening from basal 1/4 to apex; outer edge strongly sinuate; dorsal edge with S-shaped longitudinal ridge; inner edge with large, sharp tooth near 1/2 length; mesotibiae weakly curved from about 1/3 length to apex; metatibiae simple throughout its length, acutely produced apically.

*Abdomen* with five ventrites. Ventrite 1 almost as long as three following ventrites combined; ventrites 2-4 subequal in length. Ventrite 5 with lateral margins strongly converging posteriorly, posterior margin deeply, narrowly emarginate medially. Aedeagus (Fig. 3) long, heavily sclerotized, straight. Median lobe hook-shaped at apex, and branched latero-apically; branch rather long and strongly reflexed upwardly. Tegmen basal, comparatively large, ring-shaped.

#### Etymology.

The name refers to the anterior elytral macula falciform.

### Eumorphus qiujianyuei sp. n.

Taxon classificationAnimaliaColeopteraEndomychidae

http://zoobank.org/DDECE70A-4C04-4C30-BC67-5758A2CF8899

Figs 2 , 4

#### Type material.

Holotype, male, Hainan, Wuzhishan, 21.V.2014, Jian-Yue Qiu leg. (MHBU).

**Figure 2.**
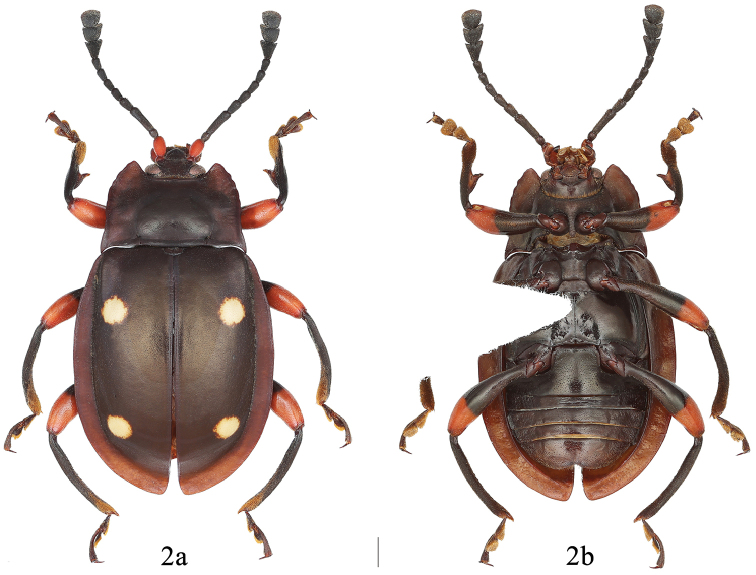
Dorsal and ventral habitus of *E. qiujianyuei* sp. n. male. **a** dorsal view **b** ventral view. Scale bar 1 mm.

#### Diagnosis.

*Eumorphus qiujianyuei* is similar to *Eumorphus austerus austerus* in appearance, but can be differentiated based on the following combination of characters: posterior angles of pronotum strongly and acutely produced, with tips curved inwardly (in *E. austerus austerus* posterior angles of pronotum weakly produced); sides of pronotum undulate (in *E. austerus austerus* rather smooth); and mesotibiae gently curved distally from near 1/2 length (in *E. austerus austerus* abruptly and strongly curved distally from near 1/2 length).

#### Description.

Length 12.3 mm. Body broadly oval, approximately 1.8 times as long as wide; moderately convex; shiny. Colour brown with four yellow maculae on elytra. Antenna with scape red. Femora at apical 1/2 or 1/3 red.

*Head*. Antenna composed of 11 antennomeres, long, rather slender, nearly 1/2 body length, with antennomeres 3–8 distinctly longer than wide; scape approximately 4.5 times as long as pedicel; pedicel short, subquadrate; antennomere 3 distinctly longer than 4−5 combined; antennomere 4 slightly longer than 5, antennomeres 5–8 subequal in length; club composed of three antennomeres, moderately broad, flat. Maxilla with terminal palpomere prolonged, nearly 2.0 times as long as palpomere 3, cylindrical, weakly curved distally.

*Thorax*. Pronotum 2.4 mm long, 4.9 mm wide; widest at base; finely, rather densely punctate; lateral and anterior margins narrowly bordered; anterior edge with small stridulatory membrane; sides undulate, distinctly converging from apical 1/3 to apex, abruptly widened basally from 1/5 length; anterior angles distinctly produced, rather acute; posterior angles strongly, acutely produced, with tips curved inwardly; disc weakly convex; median furrow absent; lateral sulci linear, deep, extending to basal ¼ length; basal sulcus nearly straight, deep. Prosternal process moderately widely separating procoxae; subparallel, weakly widening before apex then abruptly converging apically. Mesoventral process transverse rectangle, parallel sided, flat; posterior margin nearly straight. Elytra 8.9 mm long, 6.7 mm wide; 1.3 times as long as pronotum; 1.4 times as wide as pronotum, sides curved, widest near 1/2 length of elytron; lateral margins moderately widely flattened, nearly 1/5 of elytral width; distinctly converging from apical 1/3 to apex; finely, densely punctate; humeri weakly prominent. Each elytron with two small round spots. Anterior elytral spot occupies about 1/4 of elytral width, located posterior to humerus. Posterior spot of the same size as anterior one, located at apical 1/4. Protibiae in male with one large, sharp tooth near 1/2 length at inner edge, strongly expanded basally; mesotibiae distinctly curved distally from near 1/2 length; metatibiae simple, acutely produced apically.

*Abdomen* with five ventrites. Ventrite 5 with lateral margins strongly converging posteriorly, posterior margin deeply, narrowly emarginate medially. Aedeagus (Fig. 4) rather long, heavily sclerotized, weakly curved basally, abruptly widened from basal 1/3 to apex. Median lobe branched apically; the long branch abruptly raised at basal 1/3, strongly reflexed apically. Tegmen basal, comparatively large, ring-shaped.

**Figures 3–4.**
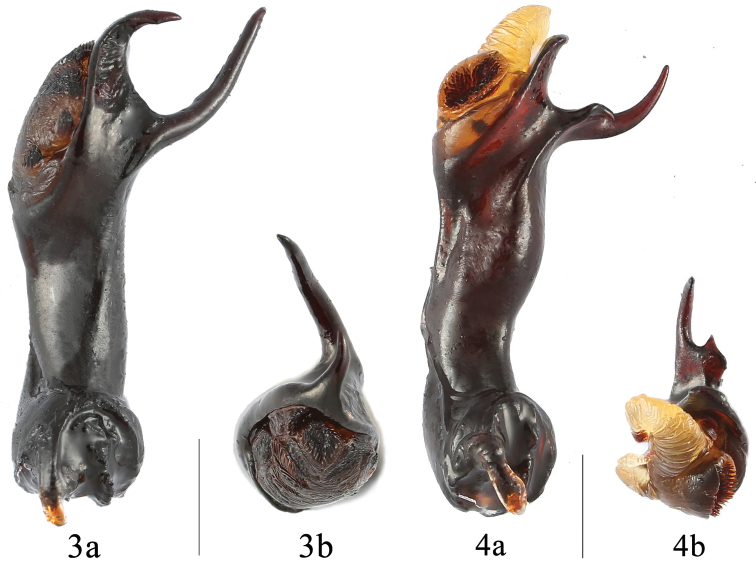
Aedeagi. **3** *E. falcifasciatus* sp. n. **4** *E. qiujianyuei* sp. n. Abbreviations: **a** lateral view **b** apical view. Scale bars 1 mm.

#### Etymology.

This new species is dedicated to Ms. Jian-Yue Qiu, an insect researcher from Chongqing, who has been working on classification of insects for many years, collecting and providing many specimens of Endomychidae used in our studies.

## Supplementary Material

XML Treatment for Eumorphus

XML Treatment for Eumorphus falcifasciatus

XML Treatment for Eumorphus qiujianyuei
